# A Multi-Camera Rig with Non-Overlapping Views for Dynamic Six-Degree-of-Freedom Measurement

**DOI:** 10.3390/s19020250

**Published:** 2019-01-10

**Authors:** Zhiyuan Niu, Yongjie Ren, Linghui Yang, Jiarui Lin, Jigui Zhu

**Affiliations:** State Key Laboratory of Precision Measuring Technology and Instruments, Tianjin University, Tianjin 300072, China; niuzhiyuan@tju.edu.cn (Z.N.); icelinker@tju.edu.cn (L.Y.); linjr@tju.edu.cn (J.L.); jiguizhu@tju.edu.cn (J.Z.)

**Keywords:** large-scale measurement, dynamic six-degree-of-freedom measurement, inside-out vision measurement, non-overlapping views

## Abstract

Large-scale measurement plays an increasingly important role in intelligent manufacturing. However, existing instruments have problems with immersive experiences. In this paper, an immersive positioning and measuring method based on augmented reality is introduced. An inside-out vision measurement approach using a multi-camera rig with non-overlapping views is presented for dynamic six-degree-of-freedom measurement. By using active LED markers, a flexible and robust solution is delivered to deal with complex manufacturing sites. The space resection adjustment principle is addressed and measurement errors are simulated. The improved Nearest Neighbor method is employed for feature correspondence. The proposed tracking method is verified by experiments and results with good performance are obtained.

## 1. Introduction

In recent years, there has been growing interest in intelligent manufacturing [1] of large-scale equipment, such as airplane assembly [2], shipbuilding [3], and spacecraft inspection [4]. As one of the key technologies in intelligent manufacturing, large-scale measurement [5] plays a crucial part in the improvement of product quality and working efficiency. Large-scale measuring instruments are expected to provide adaptive and flexible services to end-users and enable a highly integrated human-machine manufacturing system. However, popular measuring instruments like laser tracker [6], total station [7], and indoor Global Positioning System (iGPS) [8] have problems with portability and flexibility, especially in a narrow space. In practical operation, it is quite a challenging task to measure complex components with high efficiency and accuracy in a narrow ship or spacecraft cabin using any of the above instruments. Besides, operating personnel have no access to real-time visual measuring results due to the lack of interaction with measuring instruments, which makes it more difficult for them to get involved into measurement environment. Augmented Reality (AR) [9] is a novel human-machine interaction tool that combines virtual objects with real environment in a seamless way, thus offering an effective solution to large-scale measurement.

**Figure 1:**
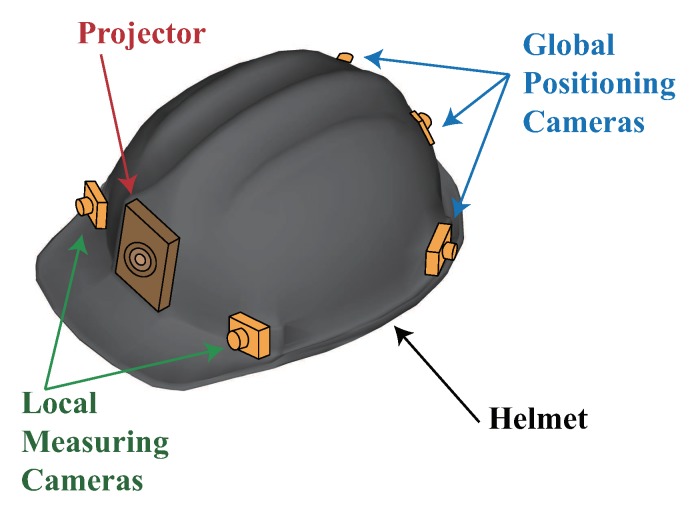
Immersive positioning and measuring helmet.

On this background, an immersive human-machine-environment interactive positioning and measuring method is proposed. By the integration of global positioning and local measuring, three-dimensional coordinates of the measured objects can be obtained in the global coordinate system. Then, based on AR, the measuring results and auxiliary information are accurately overlaid onto the measured object in real time using a projector, which enhances the user’s interactive and immersive experiences. With the immersive positioning and measuring helmet (see Figure 1), operating personnel are able to free their hands to carry out assembly and inspection work. The whole system possesses the features of high integration, excellent portability, and powerful functionality. Therefore, the immersive positioning and measuring method gives a huge boost to working efficiency with AR-assisted guidance, and it also represents the developing trend of large-scale measurement in intelligent manufacturing. In order to obtain accurate measuring results and merge virtual information with the real object perfectly, high-accuracy global positioning and tracking method is required [10]. The major task of tracking is to determine the positions and orientations of the helmet in real time, that is, dynamic six-degree-of-freedom (6-DOF) measurement [11].

A number of alternative technologies have been proposed for indoor positioning [12], such as magnetic [13], inertial [14], ultrasound [15], and vision [16]. However, complex working environment, portability, and accuracy requirement pose challenges to these methods. With small operating range, magnetic measurement is prone to distortion. Inertial measurement is of poor accuracy due to the error accumulation with time. As for ultrasound, it is severely affected by obstacles, so it does not apply to manufacturing sites. Compared with the above methods, vision measurement can realize pixel accuracy and large-scale multi-target tracking with excellent flexibility and convenience, which shows great advantages in industrial manufacturing.

The vision-based 6-DOF measurement methods can be classified into two categories: outside-in measurement [17] and inside-out measurement [18]. As for outside-in measurement, cameras are installed in the working environment and markers are fixed on the moving target. Images of markers are taken by the cameras to calculate positions and orientations of the target. The OptiTrack system [19] developed by NaturalPoint is one of the representative outside-in systems, and it produces positional error less than 0.3 mm and rotational error less than 0.05°. However, it is costly to install multiple cameras in large-scale environments, which also bring difficulties to realize exact synchronization. By contrast, inside-out measurement uses cameras mounted on the tracked object to take images of markers in the working environment, which makes it more flexible and easier to extend. As the research focus of robot autonomous navigation, Simultaneous Localization and Mapping (SLAM) [20] relies on sequences of images to recognize the robot’s location and surrounding environment. But the computational load for image correspondence is particularly high, and this view-based approach can hardly meet the accuracy requirements. In order to improve accuracy of reference points, retro reflective targets [21] are used for indoor positioning. Nonetheless, these systems lack of robustness, especially under conditions with varying illumination. Thus, active markers also have been utilized, and HiBall tracking system using LED panels is one of the most successful systems. The HiBall tracking system [22] achieves 0.5 mm and 0.03° of absolute error in a 4.5 m × 8.5 m room. However, it is quite difficult to install the LED panels at industrial spots, hindering the application of this system.

In order to better meet the needs of dynamic 6-DOF measurement for immersive positioning and measuring in manufacturing sites, this paper presents an inside-out measuring method using a multi-camera rig with non-overlapping views. The multi-camera rig is mounted on the integrated helmet for global tracking, and it is effective to increase the field of view as well as reduce the impacts of vision occlusion. Taking images of the cooperative LED markers that are deployed in the surrounding environment, positions and orientations of the helmet are determined through a collinearity equation based space resection adjustment method. As the LED markers are interchangeable with 38.1 mm spherical targets, their three-dimensional coordinates can be accurately obtained with laser tracker or industrial photogrammetry system. Furthermore, a motion information combined Nearest Neighbor (NN) method [23] is adopted to implement the matching of image points and LED markers under dynamic conditions.

The remaining of this paper is structured as follows: Section 2 describes configuration of the multi-camera rig and design of the cooperative object; Section 3 presents the dynamic 6-DOF measurement principle, including space resection adjustment method and feature correspondence method; measurement error is simulated in Section 4, while experiments are carried out in Section 5; finally, conclusions of the work are provided in Section 6.

## 2. System Hardware

**Figure 2:**
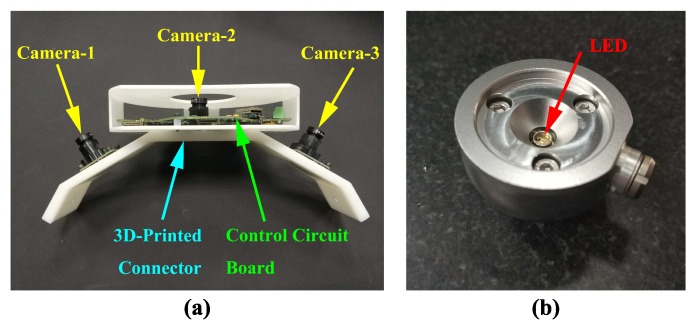
System hardware: (**a**) multi-camera rig and (**b**) active LED marker.

As depicted in Figure 2a, the multi-camera rig consists of one control circuit board and three compact CMOS cameras, which are mounted on a 3D-printed connector with good rigidity. Each camera is able to provide 1280 pixel × 960 pixel resolution with frame rate up to 54 Hz, and the size of unit pixel is 3.75 µm × 3.75 µm. As the field of view of each camera is 34.5° with a 6 mm lens, the angle between two neighboring cameras is set as 35°. Based on this design, there are none overlapping views between two neighboring cameras, so the multi-camera rig can cover larger visible range to avoid vision occlusion. The control circuit board is programmed to synchronize clocks as well as gather images, and it also transmits data to the computer via Ethernet. Consequently, the multi-camera rig is well-positioned to realize global tracking with light weight and high reliability.

The active LED markers are designed as control points to deal with complicated industrial environment. A red LED with a wavelength of 660 nm is installed at the center of a spherical target (see Figure 2b). The LED marker is aligned accurately using TESA-VISIO 300 video measuring machine. Compared with passive markers, active LED markers are less sensitive to illumination, and they can also provide optimal contrast and sharp edges. Moreover, this active LED marker is interchangeable with the 38.1 mm spherically mounted retroflector (SMR) of laser tracker.

## 3. Dynamic 6-DOF Measurement Principle

### 3.1. Camera Model

**Figure 3:**
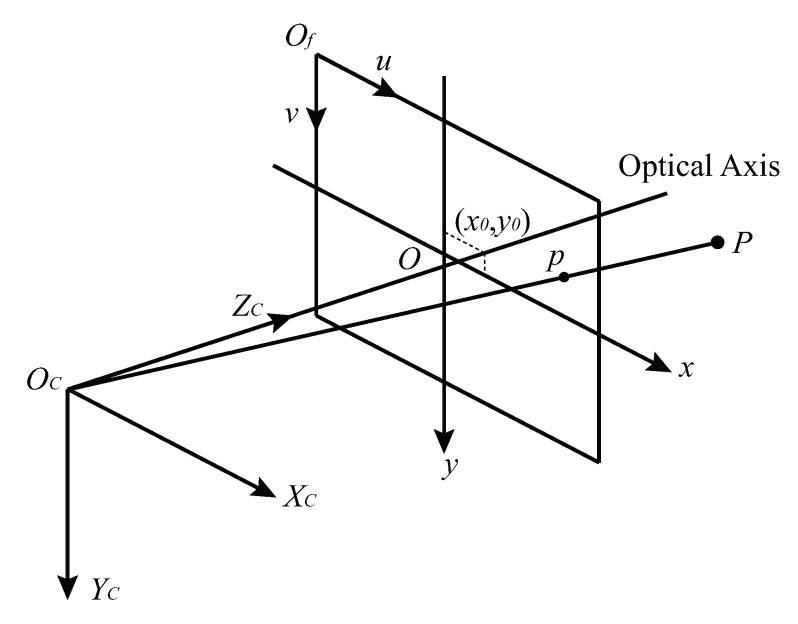
Accurate vision measurement camera model.

On the basis of pinhole model, a more complex camera model is introduced for high-accuracy vision measurement, including principal point offset and lens distortion. For convenience, the symmetrical plane of image plane is analyzed. As shown in Figure 3, a spatial object point $P(X_{p},Y_{p},Z_{p})$ is projected at $p(x_{p},y_{p})$ on the image plane through the perspective center $O_{c}$.

In the camera coordinate system ($O_{c}$-$X_{c}Y_{c}Z_{c}$), $X_{c}$ axis and $Y_{c}$ axis are parallel to *x* axis and *y* axis of the image coordinate system respectively, and $Z_{c}$ axis is along the optical axis. On account of lens installation errors, there exists an offset between the principal point $\left(x_{0},y_{0}\right)$ and the center of the image *O*. Hence, image points coordinates after principal point correction are expressed as:

(1) $$\left\{\begin{array}{cc} x_{c} & =x_{p}-x_{0}=\left(u_{p}-u_{0}\right)dx\;,\\ y_{c} & =y_{p}-y_{0}=\left(v_{p}-v_{0}\right)dy\;. \end{array}\right.$$

Here, $\left(u_{p},v_{p}\right)$ and $\left(u_{0},v_{0}\right)$ stand for pixel coordinates of point *p* and principal point respectively, and (dx,dy) are pixel separations. Besides radial lens distortion and tangential lens distortion, affine and non-orthogonality deformations also cause image point offset. The distortion is generalized into Equation (2):

(2) $$\left\{\begin{array}{cc} \Delta x & =x_{c}r^{2}k_{1}+x_{c}r^{4}k_{2}+x_{c}r^{6}k_{3}+\left(r^{2}+2x_{c}^{2}\right)p_{1}+2x_{c}y_{c}p_{2}+x_{c}b_{1}+y_{c}b_{2}\;,\\ \Delta y & =y_{c}r^{2}k_{1}+y_{c}r^{4}k_{2}+y_{c}r^{6}k_{3}+2x_{c}y_{c}p_{1}+\left(r^{2}+2y_{c}^{2}\right)p_{2}+y_{c}b_{1}\;, \end{array}\right.$$

where (Δx,Δy) denote the correction values for errors in the image plane, $x_{c}=x_{p}-x_{0}$ and $y_{c}=y_{p}-y_{0}$ stand for image point coordinates after principal point correction, $r=\sqrt{x_{c}^{2}+y_{c}^{2}}$ refers to the radical distance from image point to optical axis, $\left(k_{1},k_{2},k_{3}\right)$ represent radial distortion coefficients (generally considering the first three radial distortion coefficients), $\left(p_{1},p_{2}\right)$ stand for tangential distortion coefficients (generally ignoring the third tangential distortion), and $\left(b_{1},b_{2}\right)$ refer to affine and non-orthogonality coefficients.

(3) $$\left\{\begin{array}{cc} x_{p} & =x_{0}+\Delta x+c\frac{r_{1}\left(X_{p}-X_{0}\right)+r_{2}\left(Y_{p}-Y_{0}\right)+r_{3}\left(Z_{p}-Z_{0}\right)}{r_{7}\left(X_{p}-X_{0}\right)+r_{8}\left(Y_{p}-Y_{0}\right)+r_{9}\left(Z_{p}-Z_{0}\right)}\;,\\ y_{p} & =y_{0}+\Delta y+c\frac{r_{4}\left(X_{p}-X_{0}\right)+r_{5}\left(Y_{p}-Y_{0}\right)+r_{6}\left(Z_{p}-Z_{0}\right)}{r_{7}\left(X_{p}-X_{0}\right)+r_{8}\left(Y_{p}-Y_{0}\right)+r_{9}\left(Z_{p}-Z_{0}\right)}\;, \end{array}\right.$$

The collinearity equations are given by Equation (3) based on the camera model above, where *c* represents the principal distance, $X_{0}={\left[X_{0},Y_{0},Z_{0}\right]}^{T}$ are coordinates of perspective center in object coordinate system, and R defines rotation of object coordinates into image coordinates by three independent rotation angles θ,φ,κ about axes $X_{c},Y_{c},Z_{c}$ in Equation (4). Thus collinearity equations offer functions of six degrees of freedom $\left(X_{0},Y_{0},Z_{0},\theta ,\varphi ,\kappa \right)$ of the camera.

(4) $$\begin{array}{cc} R & =\left[\begin{array}{ccc} r_{1} & r_{2} & r_{3}\\ r_{4} & r_{5} & r_{6}\\ r_{7} & r_{8} & r_{9} \end{array}\right]\\ & =\left[\begin{array}{ccc} \cos\varphi \cos\kappa & -\cos\varphi \sin\kappa & \sin\varphi \\ \cos\theta \sin\kappa +\sin\theta \sin\varphi \cos\kappa & \cos\theta \cos\kappa -\sin\theta \sin\varphi \sin\kappa & -\sin\theta \cos\varphi \\ \sin\theta \sin\kappa -\cos\theta \sin\varphi \cos\kappa & \sin\theta \cos\kappa +\cos\theta \sin\varphi \sin\kappa & \cos\theta \cos\varphi \end{array}\right]\;. \end{array}$$

### 3.2. Feature Points Extraction Method

**Figure 4:**
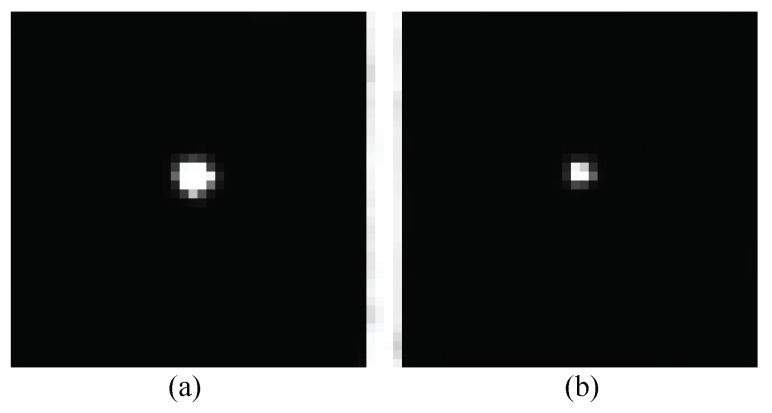
Facula of LED marker: (**a**) at a distance of 2 m and (**b**) at a distance of 5 m.

Using active LED markers as feature points, images with high contrast are acquired. The facula of LED marker is shown in Figure 4. Under this condition, the squared centroid method is adopted for sub-pixel image processing, which achieves high extraction accuracy. The squared centroid sets the gray value squared as the weight in the processing window:

(5) $$x_{m}=\frac{\sum_{ij}xf^{2}\left(x,y\right)}{\sum_{ij}f^{2}\left(x,y\right)}\;,\;y_{m}=\frac{\sum_{ij}yf^{2}\left(x,y\right)}{\sum_{ij}f^{2}\left(x,y\right)}\;.$$

Here $\left(x_{m},y_{m}\right)$ refer to the coordinates of centroid, f(x,y) is the gray value at the pixel position (x,y). Squared centroid method is computationally fast and easy to implement.

### 3.3. Space Resection Adjustment Method

**Figure 5:**
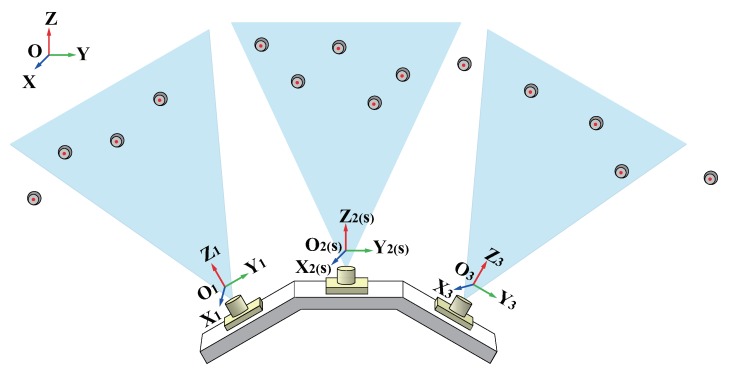
Measurement layout.

In practical measurement, a set of LED markers are deployed in the environment, as shown in Figure 5. The three-dimensional coordinates of each LED marker in the global object coordinate system *O*-XYZ are obtained. The parameters of interior orientation and spatial relationships between three cameras are also calibrated in advance,

(6) $$\left\{\begin{array}{cc} X_{1} & =R_{21}X_{2}+T_{21}\;,\\ X_{3} & =R_{23}X_{2}+T_{23}\;. \end{array}\right.$$

In Equation (6), $\left(R_{21},T_{21}\right)$ and $\left(R_{23},T_{23}\right)$ are rotation matrices and translations matrices from coordinates $X_{2}$ in $O_{2}$-$X_{2}Y_{2}Z_{2}$ to coordinates $X_{1}$ in $O_{1}$-$X_{1}Y_{1}Z_{1}$ and $X_{3}$ in $O_{3}$-$X_{3}Y_{3}Z_{3}$ respectively. Meanwhile, we assume the coordinate system of multi-camera rig $O_{s}$-$X_{s}Y_{s}Z_{s}$ is identical with the coordinate system $O_{2}$-$X_{2}Y_{2}Z_{2}$ of camera-2. Therefore, we can establish the reprojection error equations for each visible LED marker on the basis of collinearity equations:

(7) $$\left\{\begin{array}{cc} e_{xj}= & x_{j}-x_{i0}-\Delta x_{j}-c_{i}\frac{r_{i1}\left(X_{j}-X_{i0}\right)+r_{i2}\left(Y_{j}-Y_{i0}\right)+r_{i3}\left(Z_{j}-Z_{i0}\right)}{r_{i7}\left(X_{j}-X_{i0}\right)+r_{i8}\left(Y_{j}-Y_{i0}\right)+r_{i9}\left(Z_{j}-Z_{i0}\right)}\;,\\ e_{yj}= & y_{j}-y_{i0}-\Delta y_{j}-c_{i}\frac{r_{i4}\left(X_{j}-X_{i0}\right)+r_{i5}\left(Y_{j}-Y_{i0}\right)+r_{i6}\left(Z_{j}-Z_{i0}\right)}{r_{i7}\left(X_{j}-X_{i0}\right)+r_{i8}\left(Y_{j}-Y_{i0}\right)+r_{i9}\left(Z_{j}-Z_{i0}\right)}\;. \end{array}\right.$$

Here *j* is the serial number of visible LED markers and i(i=1,2,3) denotes the number of camera that observes *j*-th marker. The six degrees of freedom of camera-2 $\left(R_{2},X_{20}\right)$ with respect to the global object coordinate system can be expressed as follows,

(8) $$X_{2}=R_{2}\left(X-X_{20}\right)\;.$$

Substituting Equation (8) into Equation (6), the following relations are obtained,

(9) $$\left\{\begin{array}{cc} X_{1} & =R_{21}R_{2}\left(X-X_{20}\right)+T_{21}=R_{21}R_{2}\left(X-\left(X_{20}-R_{2}^{-1}R_{21}^{-1}T_{21}\right)\right)\;,\\ X_{3} & =R_{23}R_{2}\left(X-X_{20}\right)+T_{23}=R_{23}R_{2}\left(X-\left(X_{20}-R_{2}^{-1}R_{23}^{-1}T_{23}\right)\right)\;. \end{array}\right.$$

Therefore, the six degrees of freedom of camera-1 $\left(R_{1},X_{10}\right)$ and camera-3 $\left(R_{3},X_{30}\right)$ can be expressed by $\left(R_{2},X_{20}\right)$,

(10) $$\left\{\begin{array}{cc} R_{1} & =R_{21}R_{2}\;,\\ X_{10} & =X_{20}-R_{2}^{-1}R_{21}^{-1}T_{21}\;,\\ R_{3} & =R_{23}R_{2}\;,\\ X_{30} & =X_{20}-R_{2}^{-1}R_{23}^{-1}T_{23}\;. \end{array}\right.$$

In addition, as an orthonormal matrix, rotation matrix $R_{2}$ satisfies following constraint equations:

(11) $$\left\{\begin{array}{cc} f_{1} & =r_{21}^{2}+r_{22}^{2}+r_{23}^{2}-1=0\;,\\ f_{2} & =r_{24}^{2}+r_{25}^{2}+r_{26}^{2}-1=0\;,\\ f_{3} & =r_{27}^{2}+r_{28}^{2}+r_{29}^{2}-1=0\;,\\ f_{4} & =r_{21}r_{24}+r_{22}r_{25}+r_{23}r_{26}=0\;,\\ f_{5} & =r_{21}r_{27}+r_{22}r_{28}+r_{23}r_{29}=0\;,\\ f_{6} & =r_{24}r_{27}+r_{25}r_{28}+r_{26}r_{29}=0\;. \end{array}\right.$$

Consequently, there are only six unknown parameters, and the solution requires at least three LED markers which do not lie on a common straight line. A non-linear optimization algorithm is proposed to calculate $\left(R_{2},X_{20}\right)$, and reprojection error based object function is established as Equation (12) using Lagrange multiplier method [24].

(12) $$F=\sum_{j}^{n}\left(e_{xj}^{2}+e_{yj}^{2}\right)+\lambda \cdot \sum_{k=1}^{6}f_{k}^{2}=\min\;,$$

where *n* stands for the number of visible LED markers, λ is the Lagrange multiplier. With fast convergence rate and strong robustness, Levenberg-Marquardt (LM) algorithm [25] is employed for this optimization problem. In order to obtain global optimal solution, the initial value for optimization is calculated using EPnP algorithm [26]. Eventually, we can determine six degrees of freedom $\left(X_{s},Y_{s},Z_{s},\theta_{s},\varphi_{s},\kappa_{s}\right)$ of the multi-camera rig derived from $R_{2}$ and $X_{20}$.

### 3.4. Feature Points Correspondence Method

Because there are only several feature points that are almost exactly the same in one image, it is a huge challenge to match corresponding LED markers under dynamic conditions. NN method is proposed for feature matching by searching for the nearest point couples in two images, and each point couple represents the same LED marker. In order to improve robustness, motion information of the multi-camera rig is combined. The motion state vector of the multi-camera rig at time $t_{k}$ is $S_{k}=\left[X_{k},Y_{k},Z_{k},\nu_{xk},\nu_{yk},\nu_{zk},\theta_{k},\varphi_{k},\kappa_{k},\omega_{xk},\omega_{yk},\omega_{zk}\right]$, where $\left(\nu_{xk},\nu_{yk},\nu_{zk}\right)$ and $\left(\omega_{xk},\omega_{yk},\omega_{zk}\right)$ denote velocities and angular velocities respectively. Considering that users’ movements are normally slow, the multi-camera rig is assumed to move with constant velocities in the time Δt between two adjacent frames. Hence, the state of the multi-camera rig $S_{k}$ can be predicted by $S_{k-1}$ as follows:

(13) $$\left\{\begin{array}{cc} X_{k} & =X_{k-1}+\nu_{xk-1}\Delta t\;,\\ Y_{k} & =Y_{k-1}+\nu_{yk-1}\Delta t\;,\\ Z_{k} & =Z_{k-1}+\nu_{zk-1}\Delta t\;,\\ \theta_{k} & =\theta_{k-1}+\omega_{xk-1}\Delta t\;,\\ \varphi_{k} & =\varphi_{k-1}+\omega_{yk-1}\Delta t\;,\\ \kappa_{k} & =\kappa_{k-1}+\omega_{zk-1}\Delta t\;. \end{array}\right.$$

Then the positions and orientations of three cameras can also be predicted. Based on Equation (3), we project LED markers onto image plane and calculate image coordinates of these predicted feature points. Next, we find the nearest point couples between predicted image and real image using NN method, where the distance between two image points is defined as:

(14) $$d=\sqrt{{\left(x_{i}-x_{j}\right)}^{2}+{\left(y_{i}-y_{j}\right)}^{2}}\;.$$

In order to avoid mismatching caused by occlusion and image noise, the ratio of the shortest distance to the second-shortest distance is validated. Furthermore, the reciprocity check is employed to remove outliers. Thus the following steps need to be performed for feature points matching.

**Figure 6:**
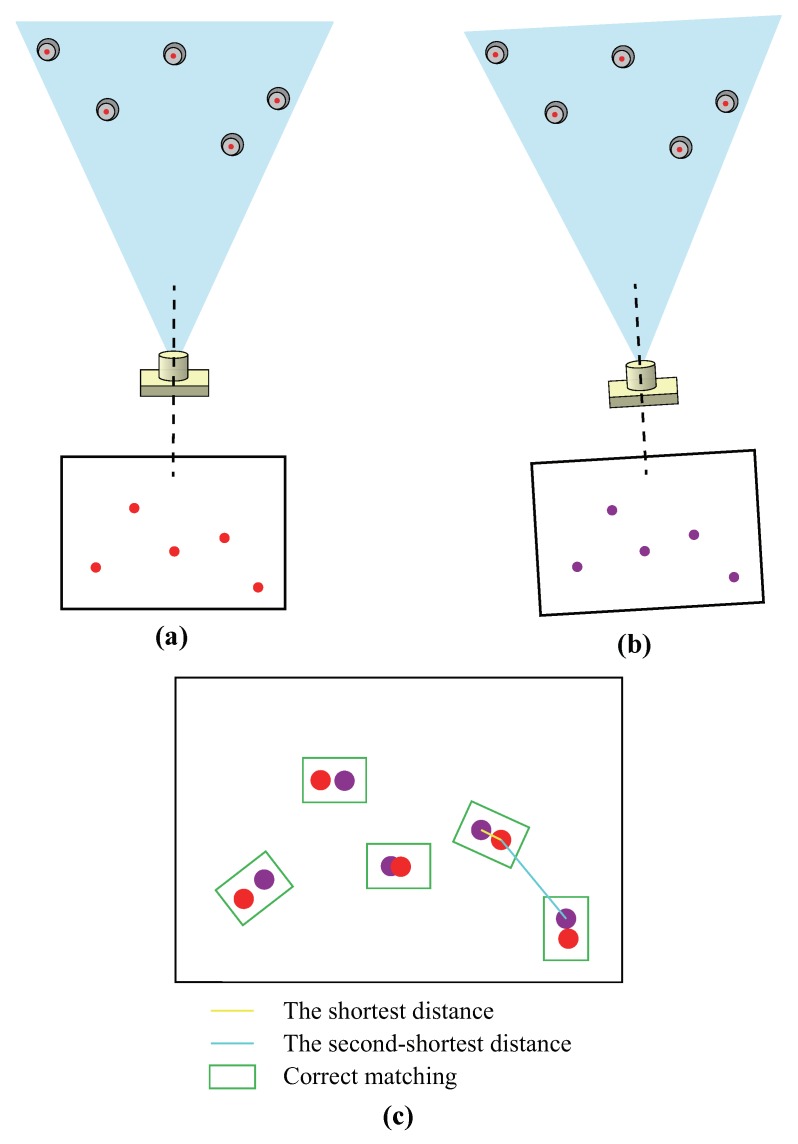
Feature points matching: (**a**) real image with real pose, (**b**) predicted image with estimated pose, and (**c**) nearest neighbor matching method.

- As for a point $P_{r,i}$ on the real image, we calculate the distances from $P_{r,i}$ to all the points on the predicted image and select its nearest neighbor $P_{p,j}$ that has the shortest distance. If the ratio of the shortest distance to the second-shortest distance is less than the threshold λ, we continue to the next step. If not, we remove the point $P_{r,i}$ as an outlier.
- We calculate the distances from $P_{p,j}$ to all the points on the real image. Then we check whether $P_{r,i}$ has the shortest distance, and whether the ratio of the shortest distance to the second-shortest distance is less than λ. When both criteria are fulfilled, the nearest point couple ($P_{r,i}$, $P_{p,j}$) are proved to be correct.
- By repeating the above process, we complete the feature points matching (see Figure 6). The value of λ is set based on the deployment of LED markers.

### 3.5. Dynamic Measurement Process

**Figure 7:**
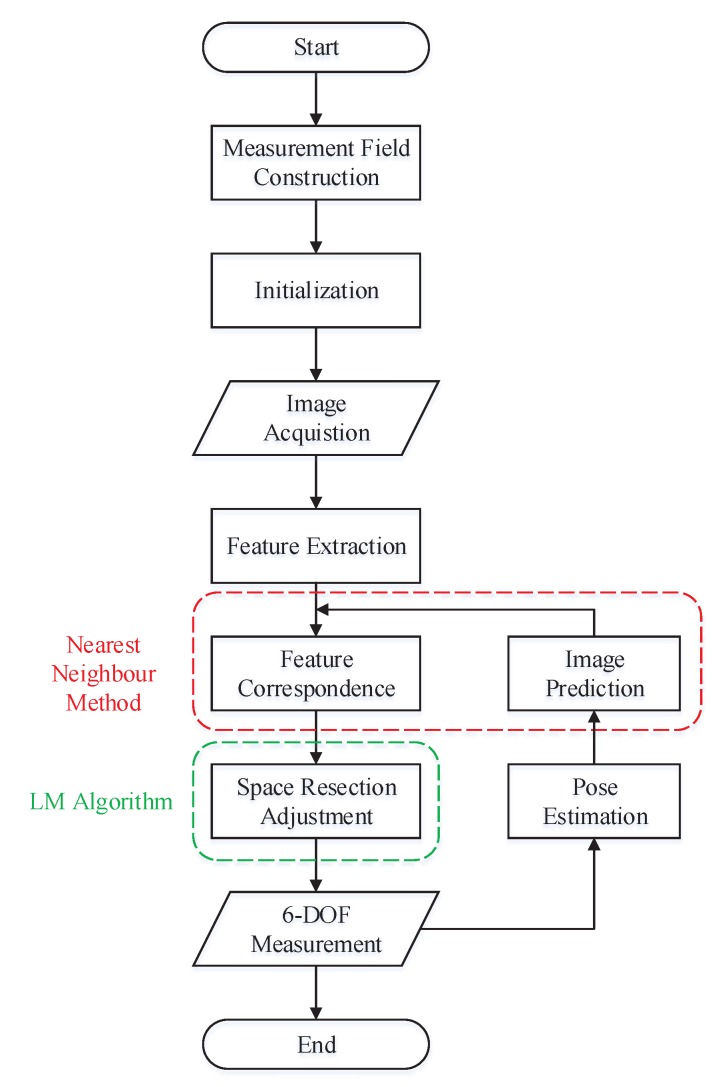
Dynamic six-degree-of-freedom (6-DOF) measurement process.

In order to accomplish continuous tracking, the system initialization has to be performed. In the initialization, the multi-camera rig remains stationary and feature points correspondence is completed manually. Once the initial state is determined, 6-DOF of the rig can be calculated real-timely in the measurement field. The complete measurement process is shown in Figure 7.

## 4. Measurement Error Simulation

On the basis of space resection adjustment method given in the previous section, measurement errors mainly arise from calibration errors of interior orientation parameters, calibration errors of spatial relationship parameters and position errors of LED markers including machining errors and measuring errors. Although measurement accuracy also depends on the focal length, the number and distribution of LED markers [27], they are not discussed in this paper.

**Figure 8:**
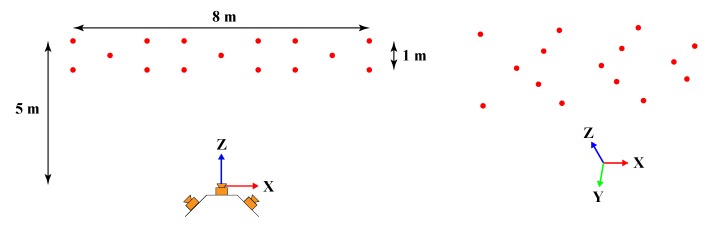
Setup of measurement error simulation.

By using Monte Carlo simulation technique, the 6-DOF measurement errors are analyzed. The deployment of the multi-camera rig and 15 markers is shown in Figure 8, and in this setup each camera observes 5 non-planar markers. The parameters of the multi-camera rig are set based on the system hardware design described in Section 2. After adding normally distributed noises, the root mean square (RMS) errors between the simulated values of 6-DOF and the true values are calculated. The following simulations are conducted to study the impacts of the above factors on the measurement errors. The sample size is set as $10^{4}$ in each simulation.

**Figure 9:**
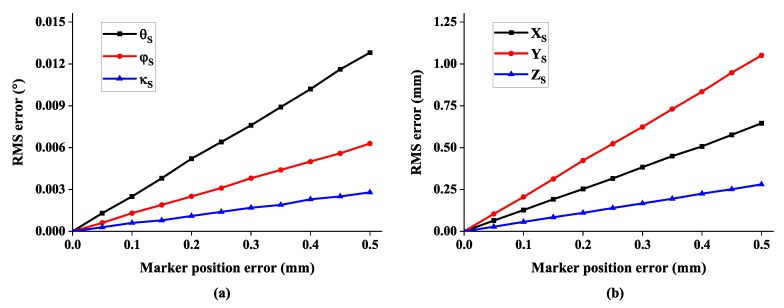
The root mean square (RMS) error of 6-DOF with respect to marker position error: (**a**) the error of rotation angle and (**b**) the error of system position.

Firstly, the impact of the marker position error is studied. The position noises obeying normal distribution are added to each marker in three axes. The standard deviation varies from 0 mm to 0.5 mm. As shown in Figure 9, the RMS error of 6-DOF increases linearly with the marker position error. When a realistic error of 0.2 mm is assumed for the markers, the three-dimensional position of the multi-camera rig is computed to an accuracy of about 0.5 mm.

**Figure 10:**
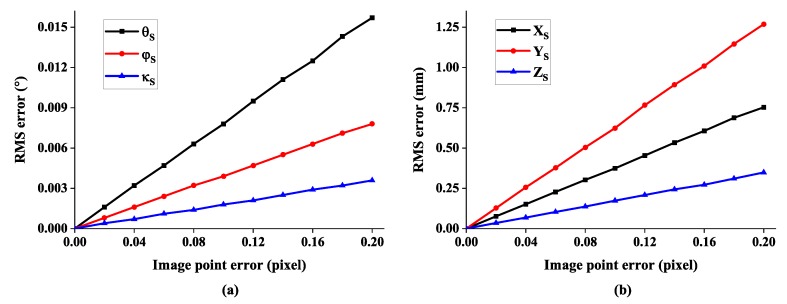
The RMS error of 6-DOF with respect to image point error: (**a**) the error of rotation angle and (**b**) the error of system position.

Since the calibration errors of interior parameters are directly reflected in the errors of image points, we add image point noises that follow normal distribution. For the simulation, the noise is altered in the range of 0.2 pixel, corresponding to 0.75 µm. Figure 10 illustrates a linear relationship between 6-DOF measurement error and image point error. With an image point error of 0.1 pixel or 0.375 µm, the angular error is less than 0.01°.

**Figure 11:**
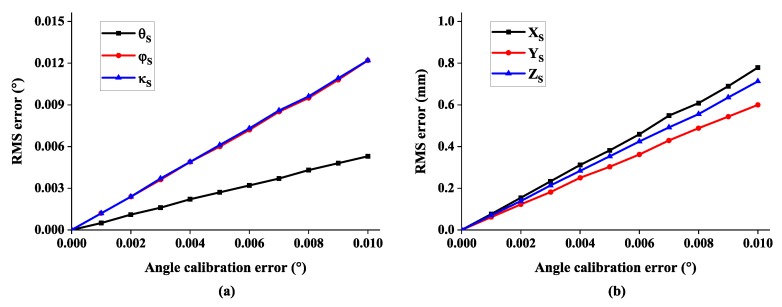
The RMS error of 6-DOF with respect to angle calibration error: (**a**) the error of rotation angle and (**b**) the error of system position.

As part of spatial relationship error, the rotation error between cameras is added to evaluate its influence on 6-DOF measurement. The noise level is changed from 0° to 0.01° and the corresponding measurement error is depicted in Figure 11. There is a clear linear trend for all six degrees of freedom. It can also be observed that the rotation angle about *X* axis is computed with better robustness than the other two rotation angles.

**Figure 12:**
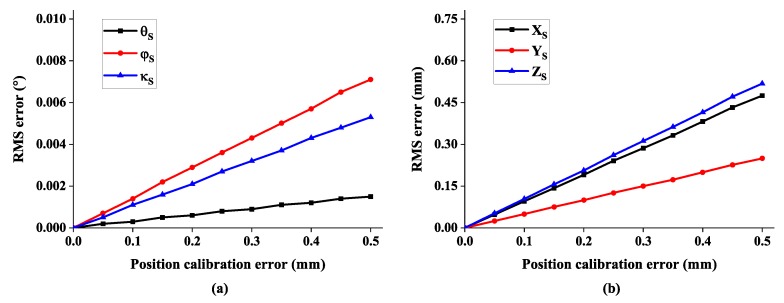
The RMS error of 6-DOF with respect to position calibration error: (**a**) the error of rotation angle and (**b**) the error of system position.

Finally, a variation of the translation error between cameras is investigated. The noise of relative position is varied within 0.5 mm. As seen from Figure 12, the 6-DOF measurement error indicates again a linear relationship to the translation calibration error [28].

Furthermore, another simulation is carried out to compare the measurement accuracy of a single camera and the multi-camera rig in the same setup above (see Figure 8). With a focal length of 2.4 mm, the single camera covers almost as wide view as the multi-camera rig to observe all 15 markers. Then the normally distributed noises including the marker position noise (0.1 mm), image point noise (0.1 pixel) and spatial relationship noise (only for the multi-camera rig) are added to simulate the 6-DOF measurement error. The spatial relationship noise is composed of the rotation noise (0.005°) and the translation noise (0.1 mm) between cameras, which are typical values for the calibration of non-overlapping cameras. The result in Table 1 shows that the multi-camera rig gives a higher accuracy than the single camera.

## 5. Experiment

Before the experiments, the multi-camera rig is fixed to a helmet. Interior orientation parameters and spatial relationship parameters of three cameras are calibrated in a large-scale spatial photogrammetric test field. Then the following experiments are conducted to evaluate the performance of the proposed method.

### 5.1. Static Measurement Experiment

**Figure 13:**
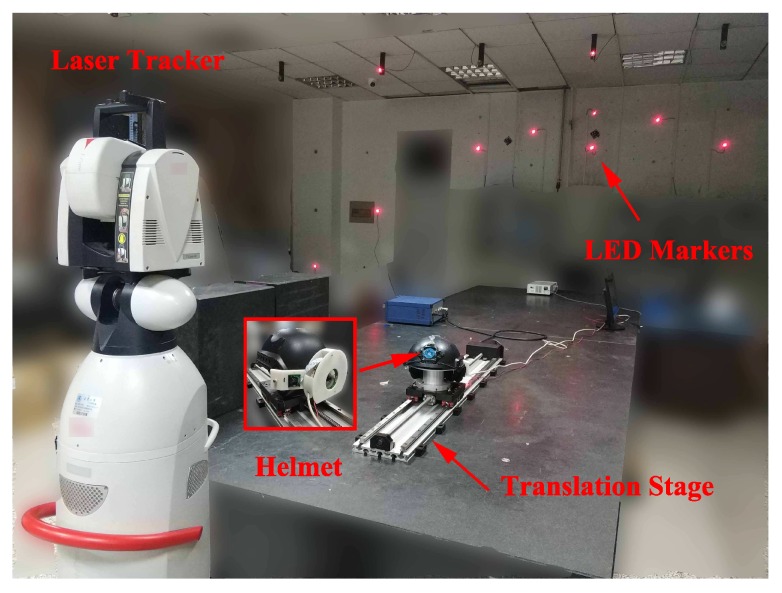
Static measurement experiment scene.

The static measurement experiment is conducted in a 5 m × 5 m × 3 m measurement field (see Figure 13). Ten LED markers are deployed, and their spatial coordinates are measured using Leica AT901 laser tracker.

#### 5.1.1. Measurement Repeatability

**Figure 14:**
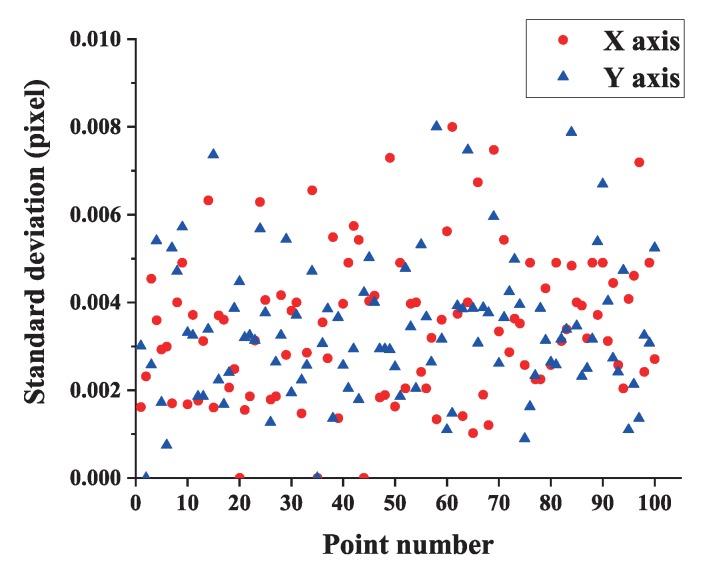
Standard deviations of image points pixel coordinates.

The helmet is randomly placed at ten different positions in the measurement field, and five images are captured at each position. Feature points are all extracted and the pixel coordinate repeatability of each point is shown in Figure 14. From the results, we can observe that the extraction precision of feature points in either axis is better than 0.01 pixel.

**Figure 15:**
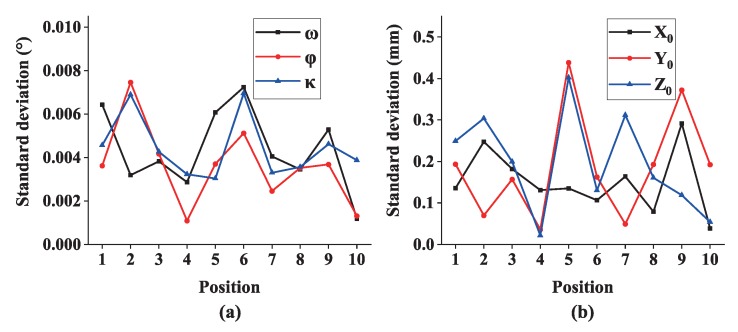
6-DOF measurement repeatability: (**a**) standard deviations of rotations and (**b**) standard deviations of positions.

Then 6-DOF $\left(X_{0},Y_{0},Z_{0},\theta ,\varphi ,\kappa \right)$ of the multi-camera rig are calculated, and 6-DOF measurement repeatability at each position is analyzed. As shown in Figure 15, standard deviations of positions along global coordinate axes are less than 0.5 mm and standard deviations of three rotations are better than 0.01°.

#### 5.1.2. Distance Measurement

The helmet is mounted on a motorized translation stage with a long travel of 1000 mm (see Figure 13), and the straightness error of the translation stage is less than 0.02 mm. The translation stage is placed in the measurement field and set to travel 900 mm each time. The positions of the multi-camera rig are obtained before and after translation, so the travel distance *D* can be calculated by $D=\sqrt{{\left(X_{a}-X_{b}\right)}^{2}+{\left(Y_{a}-Y_{b}\right)}^{2}+{\left(Z_{a}-Z_{b}\right)}^{2}}$. In addition, a SMR is fixed on the helmet to obtain accurate travel distances as reference values using the laser tracker. Nine sets of results are acquired while the translation stage is placed at nine different positions and directions, and the measurement errors of travel distances are shown in Table 2. Using the multi-camera rig, the RMS error of distance measurement is 0.383 mm.

### 5.2. Dynamic Measurement Experiment

#### 5.2.1. Operating Speed

**Figure 16:**
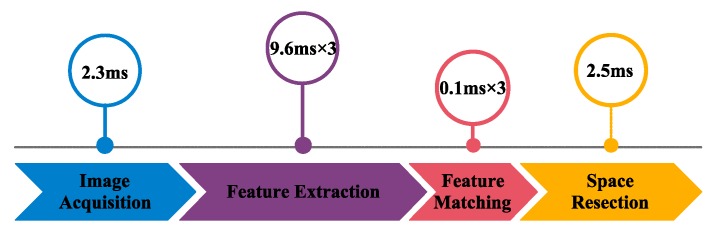
Consuming time of single measurement.

In order to evaluate the performance of dynamic measurement, the operating speed is tested using the C++ language in Visual Studio 2013 on a laptop with Inter(R) Core(TM) i7-6700HQ CPU at 2.60 GHz and 8 GB RAM. The test is conducted with ten LED markers and the maximum consuming time is shown in Figure 16. The consuming time of single measurement is approximately 33.9 ms, in which the feature extraction takes up 85% of the entire process.

#### 5.2.2. 6-DOF Measurement

**Figure 17:**
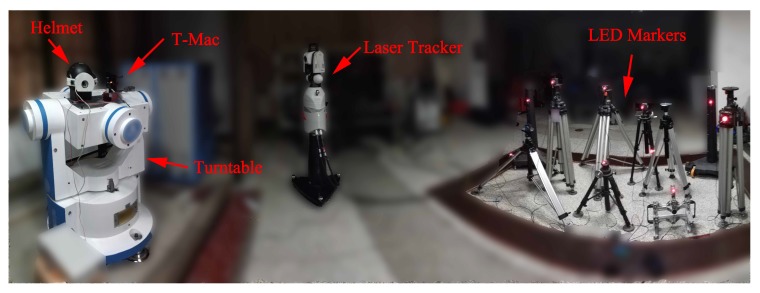
Dynamic measurement experiment scene.

As shown in Figure 17, the helmet and the Leica T-Mac are both mounted on the three-axis turntable to assess the dynamic 6-DOF measurement accuracy. Based on the experiment setup, T-Mac has a 0.01° rotational accuracy and about 30 µm positional accuracy. The spatial relationship between these two devices remains constant, no matter how the turntable rotates. Ten LED markers are deployed about five meters in front of the turntable, and their three-dimensional coordinates are measured using Leica AT901 laser tracker. In consequence, positions and orientations of the helmet and the T-Mac are unified in the laser tracker coordinate system *O*-XYZ.

**Figure 18:**
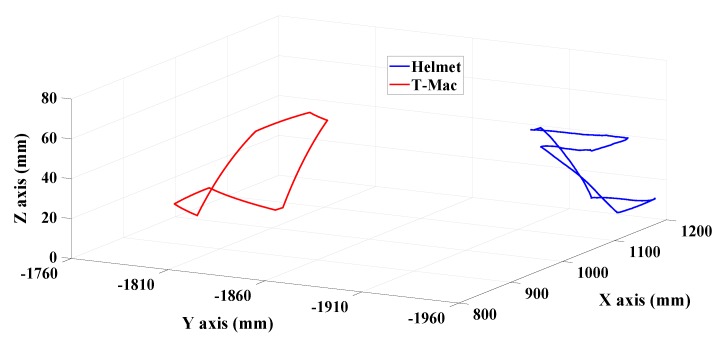
Motion trajectories of the helmet and the T-Mac in the laser tracker coordinate system.

The turntable is set to rotate 20°, 15°, and 10° about its outer axis, middle axis and inner axis respectively at an angular velocity of 5°/s, and then it returns to the starting position. In the feature matching process, NN method is applied with the threshold λ=0.3. The multi-camera rig and T-Mac are triggered at 20 Hz synchronically, and their motion trajectories are shown in Figure 18.

A test is carried out to validate the proposed feature correspondence method. Here, the sample interval is altered to simulate different angular velocities of the turntable. As for an angular velocity of 10°/s, half of the obtained images are selected with equal interval. Then feature correspondence between adjacent pictures is performed with and without motion prediction respectively. The numbers of image mismatching at different angular velocities are listed in Table 3. The results indicate that using motion prediction helps to identify better correspondences.

**Figure 19:**
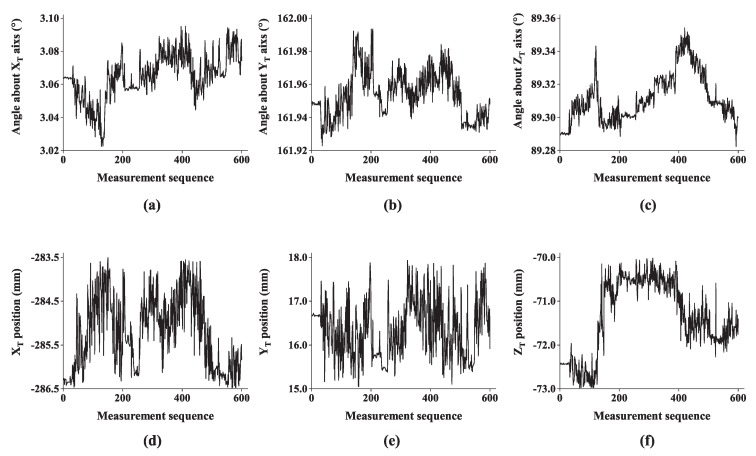
Six degrees of freedom of the helmet in the T-Mac coordinate system: (**a**) angle about $X_{T}$ axis, (**b**) angle about $Y_{T}$ axis, (**c**) angle about $Z_{T}$ axis, (**d**) position along $X_{T}$ axis, (**e**) position along $Y_{T}$ axis and (**f**) position along $Z_{T}$ axis.

Moreover, six degrees of freedom of the helmet in the T-Mac coordinate system $O_{T}$-$X_{T}Y_{T}Z_{T}$ at each triggering moment are also acquired (see Figure 19). Based on the 600 sets of data obtained, standard deviations of six degrees of freedom are listed in Table 4. Standard deviations of dynamic measurement are slightly larger than those of static measurement. This problem is probably caused by the time synchronization error for triggering the multi-camera rig and the T-Mac, which needs to be further verified.

## 6. Conclusions

In this paper, a multi-camera rig with excellent portability and high reliability is presented for dynamic 6-DOF measurement. The multi-camera rig increases the entire field of view significantly, while guaranteeing measurement accuracy by space resection adjustment. The LED markers offer a more flexible and robust solution in complex manufacturing sites. The improved Nearest Neighbor method is employed for feature correspondence under dynamic conditions. Besides, the proposed global tracking method is validated by simulations and experiments, which demonstrate good performance of static and dynamic measurement.

Considering that the proposed feature matching method is suitable for slow-moving conditions, inertial measurement unit (IMU) will be utilized in future research. With high measuring frequency, IMU provides accurate compensations of positions and orientations for vision measurement in a short time. Meanwhile, vision measurement is able to effectively correct the drift error of IMU. Therefore, vision-inertial tracking is a promising method to deal with fast and intricate movements.

## Figures and Tables

**Table 1:** Measurement accuracy comparison between the single camera and the multi-camera rig.

Parameter	The Single Camera	The Multi-Camera Rig
$\theta_{s}$ (°)	0.0147	0.0087
$\varphi_{s}$ (°)	0.0067	0.0084
$\kappa_{s}$ (°)	0.0038	0.0065
$X_{s}$ (mm)	0.715	0.629
$Y_{s}$ (mm)	1.191	0.754
$Z_{s}$ (mm)	0.292	0.528

**Table 2:** Measurement error of travel distance (mm).

Position	Multi-Camera Rig	Laser Tracker	Measurement Error
1	900.567	900.330	0.237
2	900.436	900.242	0.194
3	900.064	900.247	−0.183
4	899.970	900.381	−0.411
5	899.848	900.318	−0.470
6	899.594	900.207	−0.613
7	899.968	900.347	−0.379
8	900.588	900.185	0.403
9	900.863	900.342	0.521

**Table 3:** Numbers of mismatching with and without motion prediction

Angular Velocity	Feature Correspondence Without Motion Prediction	Feature Correspondence With Motion Prediction
5°/s	0	0
10°/s	0	0
20°/s	1	1
30°/s	6	0
40°/s	18	3
50°/s	20	7

**Table 4:** Standard deviations of dynamic six-degree-of-freedom (6-DOF) measurement

Parameter	Standard Deviation
Angle about $X_{T}$ axis (°)	0.0137
Angle about $Y_{T}$ axis (°)	0.0140
Angle about $Z_{T}$ axis (°)	0.0147
$X_{T}$ position (mm)	0.829
$Y_{T}$ position (mm)	0.640
$Z_{T}$ position (mm)	0.834

## References

[1] Zhong R.Y., Xu X., Klotz E., Newman S.T. (2017). Intelligent Manufacturing in the Context of Industry 4.0: A Review. Engineering.

[2] Jamshidi J., Kayani A., Iravani P., Maropoulos P.G., Summers M.D. (2010). Manufacturing and assembly automation by integrated metrology systems for aircraft wing fabrication. Proc. Inst. Mech. Eng. Part B J. Eng. Manuf..

[3] Lee D., Ku N., Kim T.W., Kim J., Lee K.Y., Son Y.S. (2011). Development and application of an intelligent welding robot system for shipbuilding. Robot. Comput. Integr. Manuf..

[4] Liu Y., Li S., Wang J. (2015). Assembly auxiliary system for narrow cabins of spacecraft. Chin. J. Mech. Eng..

[5] Franceschini F., Galetto M., Maisano D., Mastrogiacomo L. (2014). Large-scale dimensional metrology (LSDM): From tapes and theodolites to multi-sensor systems. Int. J. Precis. Eng. Manuf..

[6] Liu Z., Xie Y., Xu J., Chen K. Laser tracker based robotic assembly system for large scale peg-hole parts. Proceedings of the 4th Annual IEEE International Conference on Cyber Technology in Automation, Control and Intelligent.

[7] Keller F., Sternberg H. (2013). Multi-Sensor Platform for Indoor Mobile Mapping: System Calibration and Using a Total Station for Indoor Applications. Remote Sens..

[8] Schmitt R., Nisch S., Schönberg A., Demeester F., Renders S. Performance evaluation of iGPS for industrial applications. Proceedings of the 2010 International Conference on Indoor Positioning and Indoor Navigation.

[9] Fernández-Caramés T.M., Fraga-Lamas P., Suárez-Albela M., Vilar-Montesinos M. (2018). A Fog Computing and Cloudlet Based Augmented Reality System for the Industry 4.0 Shipyard. Sensors.

[10] Fang W., Zheng L., Deng H., Zhang H. (2017). Real-Time Motion Tracking for Mobile Augmented/Virtual Reality Using Adaptive Visual-Inertial Fusion. Sensors.

[11] Shi S., You Z., Zhao K., Wang Z., Ouyang C., Cao Y. (2017). A 6-DOF Navigation Method based on Iterative Closest Imaging Point Algorithm. Sci. Rep..

[12] Hassan N.U., Naeem A., Pasha M.A., Jadoon T., Yuen C. (2015). Indoor Positioning Using Visible LED Lights: A Survey. ACM Comput. Surv..

[13] Carmigniani J., Furht B., Anisetti M., Ceravolo P., Damiani E., Ivkovic M. (2011). Augmented reality technologies, systems and applications. Multimed. Tools Appl..

[14] Zhou F., Duh H.B., Billinghurst M. Trends in augmented reality tracking, interaction and display: A review of ten years of ISMAR. Proceedings of the 2008 7th IEEE/ACM International Symposium on Mixed and Augmented Reality.

[15] Nee A., Ong S., Chryssolouris G., Mourtzis D. (2012). Augmented reality applications in design and manufacturing. CIRP Ann..

[16] Bae H., Golparvar-Fard M., White J. (2013). High-precision vision-based mobile augmented reality system for context-aware architectural, engineering, construction and facility management (AEC/FM) applications. Visual. Eng..

[17] Pustka D., Hülß J., Willneff J., Pankratz F., Huber M., Klinker G. Optical outside-in tracking using unmodified mobile phones. Proceedings of the 2012 IEEE International Symposium on Mixed and Augmented Reality (ISMAR).

[18] Krum D.M., Suma E.A., Bolas M. (2012). Augmented reality using personal projection and retroreflection. Pers. Ubiquitous Comput..

[19] OptiTrack—Motion Capture Systems. www.optitrack.com.

[20] Khairuddin A.R., Talib M.S., Haron H. Review on simultaneous localization and mapping (SLAM). Proceedings of the 2015 IEEE International Conference on Control System, Computing and Engineering (ICCSCE).

[21] Mautz R., Tilch S. Survey of optical indoor positioning systems. Proceedings of the 2011 International Conference on Indoor Positioning and Indoor Navigation.

[22] Welch G., Bishop G., Vicci L., Brumback S., Keller K., Colucci D. HiBall tracker: High-performance wide-area tracking for virtual and augmented environments. Proceedings of the ACM Symposium on Virtual Reality Software and Technology.

[23] Lowe D.G. (2004). Distinctive Image Features from Scale-Invariant Keypoints. Int. J. Comput. Vis..

[24] Evtushenko Y. (1977). Generalized Lagrange multiplier technique for nonlinear programming. J. Optim. Theory Appl..

[25] Moré J.J., Watson G.A. (1978). The Levenberg-Marquardt algorithm: Implementation and theory. Numerical Analysis.

[26] Lepetit V., Moreno-Noguer F., Fua P. (2008). EPnP: An Accurate O(n) Solution to the PnP Problem. Int. J. Comput. Vis..

[27] Luhmann T. (2009). Precision potential of photogrammetric 6DOF pose estimation with a single camera. ISPRS J. Photogramm. Remote Sens..

[28] Liu Z., Zhang G., Wei Z., Sun J. (2011). Novel calibration method for non-overlapping multiple vision sensors based on 1D target. Opt. Lasers Eng..

